# Exploring the reasons why mothers do not breastfeed, to inform and enable better support

**DOI:** 10.3389/fgwh.2023.1148719

**Published:** 2023-04-12

**Authors:** Dean Roberts, Leanne Jackson, Philippa Davie, Catherine Zhao, Joanne A. Harrold, Victoria Fallon, Sergio A. Silverio

**Affiliations:** ^1^ Department of Psychology, Institute of Population Health, University of Liverpool, Liverpool, United Kingdom; ^2^ Department of Women & Children’s Health, School of Life Course & Population Sciences, King’s College London, London, United Kingdom; ^3^ Department of Nutritional Sciences, School of Life Course & Population Sciences, King’s College London, London, United Kingdom

**Keywords:** postpartum, breastfeeding, formula feeding, bottle feeding, combination feeding, social support

## Abstract

**Introduction:**

Infant and maternal breastfeeding benefits are well documented, globally. Despite efforts to increase global breastfeeding rates, the majority of high-income settings fall short of recommended targets. Breastfeeding rates in the UK are especially poor, and physiological difficulties (e.g., inverted nipples), fail to account for the observed breastfeeding intention-behaviour gap.

**Method:**

The current online study sought to investigate the infant feeding experiences of 624 UK formula feeding mothers, through open text survey responses.

**Results:**

A content analysis identified the following clusters of reasons for formula feeding: Feeding Attitudes, Feeding Problems, Mental Health, and Sharing the Load.

**Discussion:**

Feeding Attitudes explained a large percentage of reasons given for formula feeding. Recommendations are made to improve antenatal breastfeeding education and to develop an intervention with an aim to improve maternal breastfeeding attitudes and subsequent practice. Feeding Problems also explained a large portion of combination feeding and started but stopped infant feeding accounts. The current paper calls for more comprehensive and tailored antenatal breastfeeding education to refine practical breastfeeding skills necessary for successful breastfeeding establishment and maintenance. Mental Health explained relatively small coverage. Suggestions are therefore made to train mental health practitioners on infant feeding with an aim to provide more extensive support, which may serve to disrupt the bidirectional relationship between poor mental health and poor breastfeeding outcomes. Finally, Sharing the Load explained moderate coverage across never breastfed, combination fed, and started but stopped feeding groups. Recommendations are made, in light of these findings, to tighten workplace legislation to protect breastfeeding women.

## 1. Introduction

Infant and maternal breastfeeding benefits are well documented, globally (1, 2). Breastfeeding also has wide-reaching benefits at economic, social, and environmental levels within a community, creating significant national economic savings (3). The World Health Organization (WHO) draw on this extensive evidence base in recommending exclusive breastfeeding for the first six months following birth, and continued breastfeeding to two years of age and beyond (4). Ambitions have been posed to increase global six-month exclusive breastfeeding rates to 50% by 2025 (5). Currently, UK rates severely fall below official recommendations, at fewer than 1% (6). Physiological difficulties e.g., hypoplastic breasts, are unable to account for this intention-behaviour gap (7). Infant feeding decision-making is complex and determined by structural, setting, and individual level determinants (2), which will be considered in turn.

Public breastfeeding attitudes are contradictory in the UK: pro-public breastfeeding discourse is widespread across UK healthcare settings, parenting forums, and in media portrayals of infant feeding (8, 9), while simultaneously only being supported when discrete (9). Formula milk manufacturers can take advantage of maternal insecurities and proliferate misinformation with an aim to increase formula milk purchasing, which also undermines breastfeeding confidence (10). Currently, advertisement of infant foods and drinks is ineffectively regulated in the UK, allowing for aggressive formula milk marketing strategies to remain widespread in the UK (10) which has a unilateral effect on infant feeding decision-making postpartum.

Quality of health services and antenatal care influence infant feeding decision-making at a community level (2). Women who receive antenatal guidance about breastfeeding benefits were more likely to initiate breastfeeding after birth (6). Although, protective services e.g., the Healthy Child Programme (which delivers five mandatory health checks between the 28th week of pregnancy to five years postpartum (11); have suffered from reduced capacity, resourcing, and financial investment in recent years (12). Of mothers experiencing breastfeeding difficulties, 20% did not receive advice from their healthcare team, which elevated risks of early breastfeeding cessation (12). Positive breastfeeding attitudes of relatives and romantic partners facilitate breastfeeding continuation (13), while insufficient support increases the risk of early breastfeeding cessation (14). Vicarious exposure to breastfeeding within one's friendship group, too, increases likelihood of breastfeeding for an individual (6).

Personal attributes and quality of the mother-infant relationship determine postpartum infant feeding choice (2). Higher breastfeeding self-efficacy and more positively held attitudes towards breastfeeding were significantly, positively associated with breastfeeding intention and continuation (15, 16). Breastfeeding challenges are, on the other hand, notable deterrents against breastfeeding continuation (14). Most frequently reported breastfeeding challenges included: perception of poor infant feeding technique, fear of infant not receiving enough breastmilk, excessive vomiting/reflux, perceived insufficient milk-supply, positioning and latching problems, infant rejection of the breast, and painful breasts/nipples (17, 18). Perinatal mental health problems are prevalent (10%–20%) and too can negatively impact breastfeeding outcomes (19). Postnatal depression has been significantly associated with poorer breastfeeding self-efficacy, any breastfeeding status or continuation (20). The relationship between postnatal anxiety and breastfeeding outcomes follows a similar trend (21).

Nevertheless, UK breastfeeding rates have been slowly increasing in recent decades (22). Rising trends are concurrent with increasing efforts to implement Baby Friendly Initiative (BFI) standards (23). BFI adoption has been linked with improved initiation and continuation rates in international, observational studies (24). However, the mid- and long-term impacts of BFI implementation on child health outcomes in high income settings are more contested (25). BFI-informed care has been criticised for overlooking sociocultural and structural barriers (26) and for promoting unrealistic breastfeeding expectations which manifest feelings of guilt and disappointment (26).

Collectively, one-dimensional promotional models appear ineffective for optimising breastfeeding outcomes, when compared with individualised initiatives and multicomponent interventional efforts (25, 27, 28). Pre-existing promotional strategies have also been ineffective in supporting breastfeeding continuation in line with WHO guidelines.

## 2. Materials and methods

The current study sought to understand, in greater depth, the infant feeding experiences and difficulties of women in the United Kingdom, in their first six months postpartum. The online survey was advertised via social media and online parenting forums and was completed by mothers (*N* = 624, *M_Age _*= 29.44 years) of infants (*M_Age _*= 17.96 weeks) who: Never Breastfed (NB, *n* = 158); Started, But Stopped Breastfeeding (SBS, *n* = 278); and who Combination Fed (CF, *n* = 188). We used mothers' qualitative responses to the question: “*What were the main reasons you chose to formula feed your baby?”* to address the following aims: (a) To investigate reasons given for not breastfeeding; and (b) To illuminate commonalities in reasoning across feeding groups.

The study received ethical approval from the University of Liverpool Institute of Psychology, Health and Society Research Ethics Committee (ref:- IPH/2047). All participants provided consent to participate on the first page of the on-line survey.

We utilised a qualitative content analysis (29) to allow for the content of this heterogenous textual data to be codified using a systematic process of categorisation, to produce thematic clusters derived from the text which can then be interpreted.

In the first organising phase: open coding, classification, and abstraction were undertaken, where two researchers were responsible for the creation of possible thematic clusters. These were labelled and categories were organised into these thematic clusters. This was followed by the coding phase, where the textual excerpts were read numerous times before being assigned to the appropriate thematic cluster, and coding were then categorised (i.e., all codes were assigned to thematic clusters). Finally, thematic clusters were assessed for thematic overlap and those clusters which were similar were made into broader clusters to minimise the number of overall thematic clusters. The presentation of results is always the final step in content analysis, which we have provided in a tabular format.

Coding and analysis were consultative whereby if different coding was identified, comparisons were made and researchers worked to compromise over nuance and semantic differences. Another researcher would arbitrate if agreement could not be reached.

The final sample of this qualitative analysis included 624 mothers. Frequencies were calculated to provide the number of occurrences of a particular code within the responses across all thematic clusters, and results were stratified by the three participant groups (NB; SBS; CF) to allow for comparison and observation of results across all participants. A proportion of our final sample (12.55%) did not provide open text responses or gave illegible responses e.g.,: “*choose”,* which were excluded from analysis.

## 3. Results

All themes identified during the analysis of 624 respondents are presented in Table 1. Infant feeding experiences and difficulties reported by respondents were sorted in to four thematic clusters: Feeding Problems; Mental Health; Feeding Attitudes; and Sharing the Load. Thematic clusters were split by infant feeding method: Never Breastfed (NB); Started but Stopped Breastfeeding (SBS); and Combination Feeding (CF).

**Table 1:** Clusters of themes identified from content analysis, with provided examples in the form of statements and percentages.

Coding cluster	Participant group	Example Quotations	Frequency of occurrence
Feeding problems	NB	“I tried to feed my first baby but had latching difficulties. I then turned to formula…” (Participant 1) “Inverted nipples mean baby can't latch on. I expressed milk for my first baby but this time found that I wouldn't have enough time.” (Participant 24)	32.97%
	SBS	“I started off breastfeeding, but my baby never seemed happy. Changed to formula feeding at 6 weeks.” (Participant 50)	69.81%
	CF	“I have never got the hang of expressing so if she stays with family, she has formula.” (Participant 37) “Insufficient supply of breastmilk. Improper latch as time with lactation consultant was insufficient after birth…” (Participant 10)	67.59%
Mental health	NB	“Postnatal depression after problems with breastfeeding the 1st child.” (Participant 98)	4.86%
	SBS	“My mental health and caring for my child became more important than him having breast milk.” (Participant 53) “I suffered really bad postnatal depression, so being able to just up and leave if I needed to get away for a few mins was a plus!” (Participant 177)	5.84%
	CF	“I didn’t feel strong enough (mentally, emotionally or physically) to persevere with exclusively breastfeeding” (Participant 54)	1.39%
Feeding attitudes	NB	“Repulsed by the idea of lactating” (Participant 17)	32.97%
	SBS	“My daughter was mostly formula fed and has turned out amazing. She's very bright, rarely gets sick, and not overweight… actually is much healthier than her breastfed friends” (Participant 104)	4.87%
	CF	"Bad experience from the first child when trying to breastfeed led me to start with both this time” (Participant 3)	4.63%
Sharing the load	NB	“I concluded that if wouldn't have the time to both focus on building a solid breastfeeding relationship with the new baby whilst giving my 2 year old all the care and attention she needed/deserved…” (Participant 43) “I had to go back to work very quickly after giving birth. I felt there was no point in trying to get breastfeeding to work if someone else was just going to have to give my baby a bottle.” (Participant 60)	16.22%
	SBS	“I found it difficult to be relied on 100% of the time by baby and felt tied to her and unable to go out…” (Participant 155)	8.77%
	CF	So my partner could share feed times and feel closer to his baby girl…” (Participant 45)	12.04%

### 3.1. Feeding problems

For CF mothers, 67.59% reported feeding difficulties. Infant feeding difficulties centred around perceived insufficient milk supply, poor latching technique, and practical difficulties expressing breastmilk. For SBS mothers, 69.81% also articulated feeding problems, though for these women difficulties were more infant-focused, describing infant as being dissatisfied with breastmilk which led to early breastfeeding cessation. Feeding difficulties were reported by 32.97% of NB mothers, also.

### 3.2. Mental health

Coverage for mental health difficulties among SBS mothers was minimal with only 5.84% reporting data coded within this thematic cluster. For these women, postnatal depression ensued following experience of stubborn breastfeeding challenges. 4.86% of NB mothers reported mental health difficulties. For these women, the convenience and flexibility which formula milk provided with regards to allowing the mother to engage in self-care activities and to care for her infant outweighed the benefits of breastfeeding. Mental health difficulties were reported by 1.39% of CF mothers, also.

### 3.3. Feeding attitudes

There was minimal coverage of feeding attitudes by CF mothers with just 4.63% reporting, and likewise for SBS mothers who had 4.87% reporting frequency. Reasons provided for poor feeding attitudes included negative experiences of breastfeeding attempts with older children and holding positive attitudes about the development of the mother's older, formula fed children, respectively. However, NB mothers reported more negative attitudes towards breastfeeding with 32.97% recalling data covered by this thematic cluster. For these women attitudes were held more strongly about the idea of breastfeeding, conceptually.

### 3.4. Sharing the load

CF and SBS mothers expressed sharing the load as reasons for their infant feeding method, with 12.04% and 8.77% reporting data, respectively. More NB mothers reported on this theme, at 16.22%. NB mothers noted formula feeding allowed the mother to balance infant care responsibilities more easily with parenting responsibilities for older children and with employment-based responsibilities**.**

## 4. Discussion

### 4.1. Summary of findings

A content analysis was conducted on open text, online survey responses collected from 624 from NB, SBS, and CF mothers. The content analysis identified four themes pertaining to reasons given for formula feeding method, which were: feeding problems, mental health, feeding attitudes, and sharing the load.

Feeding problems had comparatively large percentage coverage in reasons provided for formula feeding method. This was observed across CF and SBS groups, with mothers specifically commenting on difficulties establishing a successful breastfeeding latch. These findings parallel previous literature, which also reports practical breastfeeding difficulties, such as unsuccessful latching, to be a primary reason given for early breastfeeding cessation (17, 30–33). Previous literature has shown that professional prenatal breastfeeding education can increase latch skills, reduce nipple damage during breastfeeds (34), and extend breastfeeding duration (though methodological heterogeneity and poor research quality contribute towards mixed findings, and limits the ability to form firm conclusions (35). This suggests that specialised maternal support may serve to acknowledge and address breastfeeding issues, reinforcing calls for action reported in pre-existing infant feeding literature (36).

Unsurprisingly, those who NB did not disclose feeding problems. Formula feeding is an attractive infant feeding option for mothers with busy lifestyles (37). However, current findings demonstrate that a proclivity to breastfeed may be sourced in insufficient knowledge, supporting the notion that breastfeeding is a learned skill (38). Feeding problems are inherently linked with poorer understanding and refinement of breastfeeding skills (39, 40). Comprehensive and tailored education and support throughout pregnancy and the postpartum might deem breastfeeding a more viable option for new mothers.

Mental health was an underlying reason for formula feeding among all infant feeding groups. Specifically, formula feeding alleviated depressive symptoms for mothers in the current and in previous studies (41). This finding is concurrent with previous trends identifying a relationship between postnatal depression and poor breastfeeding exclusivity and duration (20, 42). Depressive symptoms disrupt the production of hormones involved in breastfeeding e.g., milk ejection reflex (43–45). Conversely, breastfeeding significantly increases levels of oxytocin and subsequent emotional recognition (46), which is important for emotional processing of stress, anxiety, and for sensitivity to infant affect (47). Improving accessibility to postnatal mental health support could act as a circuit break in the bidirectional relationship between maternal mental ill-health and poor breastfeeding outcomes (48–50), and may serve to improve both maternal emotional wellbeing and breastfeeding outcomes. Providing mental health providers with infant feeding education results in the provision of better tailored psychological support (49), which is a key recommendation of this paper.

Feeding attitudes were also proportionately large predictors of formula feeding status among all three groups. Positive breastfeeding attitudes being held by relatives and partners of the mother facilitate breastfeeding continuation (51), whereas negatively held attitudes can pose as notable barriers to successful breastfeeding practice (52). Infant feeding attitude held during pregnancy has shown stability over time, predicting breastfeeding initiation, duration, and exclusivity (53). In the current study, negative breastfeeding experiences with previous children averted mothers from attempting breastfeeding with their youngest infant, consistent with previous literature (54).

Feeding attitudes were an especially notable predictor for NB mothers. Pro-formula feeding attitudes predict exclusivity more so than knowledge of breastfeeding benefits (55), and evidence suggests that formula feeding women commonly hold misconceptions about breastfeeding (56). Among formula feeding women, previous breastfeeding experiences significantly predicted breastfeeding initiation and duration in subsequent births (57) and more positive formula feeding attitudes predicted formula feeding intention during pregnancy (56). Feeding attitudes are malleable (53), and intervention-based studies have shown utility in improving breastfeeding attitudes and postpartum outcomes (58).

On the topic of intervention research—morally charged promotional breastfeeding discourse can unintentionally cultivate feelings of guilt and shame for those who cannot, or do not breastfeed (59, 60). It is therefore important for intervention efforts to remain mindful of potential ramifications of “*breast is best*” discourse, and to adopt an incremental goal setting approach to behaviour change (61). Current findings also support the notion that formula feeding attitudes and breastfeeding attitudes are not antagonistic, but rather independent (62). Positive formula feeding attitudes are predictive of breastfeeding cessation (63). Targeting positively held attitudes towards formula milk, over promoting positive attitudes towards breastmilk, is a potential avenue for intervention (10).

The final major theme, sharing the load, was cited by mothers across all feeding methods, but was especially pronounced among NB mothers. Breastfeeding is a resource-taxing infant feeding method which places sole caregiving responsibility on the mother (64). Breastfeeding, therefore, can be especially difficult for mothers balancing conflicting social identities e.g., balancing childcare and work responsibilities (2, 65). Practical support from one's maternal grandmother (66), romantic partner (67), and from one's employer (68, 69) can ease the perceived demands of breastfeeding. Consequently, sharing the load may reflect perceived insufficient support from one's social support network and/or insufficient advocation of one's needs early postpartum. In the current study, sharing the load encompassed difficulties in finding private space(s) to breastfeed and struggling to manage infant feeding demands and work responsibilities (70). Although workplace protection exists for breastfeeding women (71) which positively impacts breastfeeding outcomes for working mothers (72), employer adherence to these guidelines is mixed (2, 73). Greater adherence to the WHO code on Marketing of Breastmilk Substitutes (10), which, among other legislative forms of protection, mandates breastfeeding employers are reasonably supported in returning to work while nursing e.g., flexible working hours, implementation of expression rooms, might serve to improve breastfeeding outcomes for working mothers.

### 4.2. Strengths limitations, and future research

The methodological framework adopted in the current study allowed for formula feeding participants to share candid responses regarding the often ‘taboo’ subject of breastfeeding cessation (59, 60). It was important that this element of social desirability was controlled, in a demographic where feeling inadequate and dejected socially is commonplace (74). A sampling bias exists in perinatal literature, whereby the majority of participants tend to be exclusive or partial breastfeeders (59). Online data collection enabled a large sample of formula feeding women to be recruited, comparable to previously published infant feeding quantitative works (75). However, the open text response format in the survey lacked control over quality of respondent answers, with some participants providing vagaries. In the current study, women self-identified their formula feeding status. Reliance on self-identification in some instances led to discrepancies with researcher understanding of infant feeding categories, which may have led to misclassification e.g., a proportion of our sample self-identified as NB, while in-text they reported having given one breastfeed postpartum. Self-identification is, however, paramount in one's interpretation of events (76). Gaining the insights and perceptions of these women was essential for addressing study aims, warranting the self-reported data collection method. Furthermore, due to the self-selecting nature of the research, it could be possible that participants with negative experiences presented to the research, whereas those with neutral or positive experiences of breastfeeding may have chosen not to participate. Within the current methodological design, reasons for breastfeeding cessation were recorded retrospectively, meaning that may have increased chances of response bias.

### 4.3. Conclusions

The current study used content analysis on open text, online survey responses collected from 624 NB, SBS, and CF mothers to address the following aims: (a) To investigate reasons given for not breastfeeding, and (b) To illuminate commonalities in reasoning across feeding groups. Feeding problems explained a large percentage coverage in reasons for formula feeding. The current study recommends comprehensive prenatal breastfeeding educational programmes to address feeding difficulties commonly experienced during the early postpartum and to encourage those with formula feeding intent to consider breastfeeding as a viable infant feeding option. Improving accessibility to and quality of perinatal mental health support services may serve as a circuit break for the bidirectional relationship between maternal mental ill health and poor breastfeeding outcomes. Interventions are proposed, which adopt an incremental goal setting approach to breastfeeding, and are recommended to alter breastfeeding attitudes more favourably for formula feeding mothers. Finally, tighter legislation on workplace protection of lactating mothers and active encouragement of the maternal family unit's support and advocation of breastfeeding practice are recommended to support women who wish to breastfeed.

## Data Availability

The raw data supporting the conclusions of this article will be made available by the authors, without undue reservation.
